# Assessment of the visual pathways in patients with neurofibromatosis-1 by 3S-space technique with 3-Tesla MRI

**DOI:** 10.3906/sag-1906-14

**Published:** 2019-12-16

**Authors:** Özge SARAÇ, Oktay ALGIN, Mehmet BEYAZAL, Banu ANLAR, Ali VARAN, Tülay KANSU

**Affiliations:** 1 Department of Ophthalmology, City Hospital, Yıldırım Beyazıt University, Bilkent, Ankara Turkey; 2 Department of Radiology, City Hospital, Yıldırım Beyazıt University, Bilkent, Ankara Turkey; 3 National MR Research Center (UMRAM), Bilkent University, Bilkent, Ankara Turkey; 4 Department of Radiology, Faculty of Medicine, Recep Tayyip Erdoğan University, Rize Turkey; 5 Department of Pediatric Neurology, Faculty of Medicine, Hacettepe University, Sıhhıye, Ankara Turkey; 6 Department of Radiation Oncology, Faculty of Medicine, Hacettepe University, Sıhhıye, Ankara Turkey; 7 Department of Neurology, Faculty of Medicine, Hacettepe University, Sıhhıye, Ankara Turkey

**Keywords:** Neurofibromatosis type 1, optic pathway glioma, 3D-SPACE, optic nerve thickness, optic nerve sheath diameter, optic nerve tortuosity

## Abstract

**Background/aim:**

We aimed to evaluate the size/tortuosity of the optic nerve (ON) and the dilatation of the ON sheath (ONS) in neurofibromatosis type 1 (NF-1) patients with 3T-MRI, and to assess the usefulness of 3D-SPACE in imaging the optic pathway, ON, and ONS in NF-1 patients.

**Materials and methods:**

Twenty consecutive NF-1 patients without optic pathway glioma (OPG) (Group 1), 16 consecutive NF-1 patients with OPG (Group 2), and 19 controls were included in this study. The thickness and tortuosity of the ON and the diameter of the ONS were measured on STIR and 3D-SPACE images.

**Results:**

The thickness of the ON was similar in all groups on STIR images (P>0.05). The mean ONS diameter was higher in Group 2 with this sequence (P=0.009). Controls had significantly lower grades of ON tortuosity than Groups 1 and 2 (P=0.001), and Group 1 had significantly lower ON tortuosity compared to Group 2 (P=0.001). Severe tortuosity was only detected in Group 2.

**Conclusion:**

ON tortuosity and ONS diameter were increased in NF-1 patients in the presence of OPG. High-resolution cranium imaging with the 3D-SPACE technique using 3T-MRI seems to be helpful for detection of the optic pathway morphology and pathologies in NF-1 patients.

## 1. Introduction

Neurofibromatosis type 1 (NF-1) is an autosomal dominant neurocutaneous disorder characterized by multiple neurofibromas, cutaneous pigmentation, and skeletal dysplasia [1]. Individuals with NF-1 typically present in childhood with café-au-lait spots, inguinal and axillary freckling, and Lisch nodules on the iris [2]. Numerous central nervous system (CNS) manifestations have been identified in NF-1 including optic pathway gliomas (OPGs), optic nerve tortuosity, cerebral astrocytomas, hydrocephalus, and neurofibromatosis bright objects (NBOs) on imaging [3].

OPGs are the most common CNS neoplasms in NF-1, encountered in 15% of NF-1 patients [1,4]. They generally involve the anterior visual pathway. Although 45% are intraorbital, they can arise from anywhere else in the visual pathway including the optic chiasm, optic tracts, and optic radiations [1]. The last location is relatively rare but exhibits more aggressive behavior [4–8].

Magnetic resonance imaging (MRI) is the method of choice for detection and evaluation of OPGs and other intracranial manifestations of NF-1 because of its higher soft-tissue contrast resolution and radiation-free multiplanar imaging capability [9]. Many institutions perform MRI screening of the brain in all young patients with NF-1 and chiasm with thin slices through the optic nerves, and some authors argued that they only perform MRI when a reliable eye exam cannot be obtained or there are other clinical abnormalities [1,6–8]. As a result, many patients with NF-1 undergo MRI during childhood. In certain cases, multiple MRIs including brain, orbital, and/or phase-contrast cine are performed for accurate demonstration of the CNS lesion [8]. Multiple imaging examinations have their own disadvantages, such as lower patient cooperation and comfort, higher specific absorption rate (SAR) level, and increased imaging time/cost [10]. New achievements in MRI technology allow the entire cranium to be scanned three-dimensionally (3D) in thin sections within 5 min by 3D sampling perfection and application-optimized contrasts using the different flip-angle evolutions (3D-SPACE) technique [11]. The 3D-SPACE technique has the capacity to determine all CNS manifestations of NF-1 patients and give additional information undetectable with routine MRI sequences in clinical settings. 

Optic nerve (ON) tortuosity and ON sheath (ONS) dilatation are abnormalities of patients with NF-1 [8]. They generally are identified incidentally. Their significance in the prognosis of NF-1 patients is not known in current clinical practice yet. The incidence of ON tortuosity in patients with NF-1 was between 12% and 31% in previous studies [8,12].

We evaluated the optic pathways of NF-1 patients with and without OPG with a 3-Tesla (3T) MRI unit. In this study we had two aims: first, to assess the relationship between the presence of OPG and the thickness/tortuosity of the ON and the diameter of the ONS, and second, to investigate the significance of 3D-SPACE sequences in the assessment of the optic pathway of NF-1 patients using a 3T-MRI unit. To our knowledge, this is the first study evaluating the optic pathway with 3D-SPACE sequences in NF-1 patients using 3T-MRI.

## 2. Materials and methods

This prospective study was conducted in compliance with institutional and government review board regulations, informed consent regulations, and the Declaration of Helsinki during a 3-year period. Written informed consent was obtained from all patients and control subjects before the clinical and radiological evaluations. 

Twenty NF-1 patients without OPG (Group 1), 16 NF-1 patients with OPG (Group 2), and 19 healthy subjects with normal neurological and ophthalmological examinations (control group) were enrolled in this study. Subjects with any history of neurological disease, allergy, claustrophobia, ferromagnetic implants or pacemakers, renal failure, meningitis, and pregnancy were excluded. In five cases, screening failures occurred. These cases were also excluded from the study. The presence or absence of OPG was defined subjectively by an experienced neuroradiologist (O.A.) according to the literature [1–8]. Presence of headache was also subjectively questioned and recorded. 

The diagnosis of NF-1 was made according to the National Institutes of Health Consensus Development Conference diagnostic criteria [1]. After the neuroophthalmological examination, 3T-MRI acquisitions were obtained from all subjects. A neuroradiologist (O.A.) evaluated the MRI data during acquisition; if needed, intravenous contrast medium (0.3 mL/kg Dotarem) was administered before the 3D-SPACE and STIR sequences and postcontrast T1W images were obtained. MRI protocol is given in Table 1. MRI examinations were performed using a 3T unit (Trio with Tim; Siemens Healthcare AG, Erlangen, Germany) with a birdcage multichannel head coil.

**Table 1:** 3-Tesla MRI protocol used for the study.

Sequences/ Parameters	3D-MPRAGE	3D-SPACE (with VFAM)	STIR	FLAIR
TR/TE (ms)	2130/3.45	3000/579	5100/81	6000/405
TI (ms)	1100	-	150	2100
Slice thickness (mm)	0.8	0.6	3	0.9
FOV* (mm)	230 × 230	240 × 240	230 × 230	230 × 230
Acquisition time (min)	5	5	4	9
NEX	1	2	2	1
Number of slices	240	240	16	192
Flip angle (°)	8	100	120	-
Imaging plane	Sagittal	Sagittal	Axial-coronal	Sagittal
Distance factor	-	-	10%	-
PAT factor	2	2	-	--
PAT mode	GRAPPA	GRAPPA	-	-
Voxel size (mm)	0.8 × 0.8 × 0.8	0.6× 0.6 × 0.6	0.6 × 0.5 × 3	0.9 × 0.9× 0.9
FA mode	-	T2 variant	-	-

TI: Time of inversion; 3D-SPACE: three-dimensional sampling perfection with application-optimized contrasts using different flip angle evolutions; 3D-MPRAGE: 3D T1W magnetization prepared rapid acquisition gradient-echo; STIR: short tau inversion recovery; FLAIR: fluid-attenuated inversion recovery; NEX: number of excitations; FOV: field of view; PAT: parallel acquisition technique; GRAPPA: generalized autocalibrating partially parallel acquisitions. The advantage of 3D acquisition is the capability to reconstruct the images at any angle and align with other acquisitions. Thus, multiplanar images were obtained from 3D-MPRAGE, SPACE, and FLAIR acquisitions.

**Figure 1:**
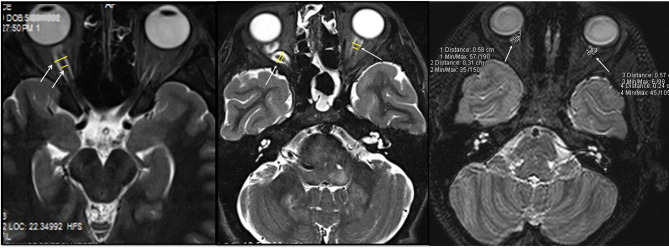
Optic nerve (ON) and optic nerve sheath (ONS) measurements (arrows) of 3 patients with NF-1 on STIR (left and middle) and 3D-SPACE (right) images.

Multiplanar and curved reformatted images were obtained with Neuro 3D software (Siemens Healthcare AG, Germany), except the STIR sequence, because routine STIR is a 2D sequence. The thickness of the ON and the diameter of the ONS were measured on short tau inversion recovery (STIR) and 3D-SPACE images by another neuroradiologist (M.B.). For each eye, two measurements were made from the thickest part of the ON and ONS, which did not involve the qualitatively (visually) detectable part of the OPG (Figure 1). The mean value of these two measurements was calculated and considered as the exact value of each eye. The measurements were made subjectively by each rater in a uniform manner between outer margins on axial and coronal images. The rater (M.B.) was blinded to the patient group during the evaluations. All measurements were made for the intraconal thickest part of the ON for both eyes separately. If the ON and/or ONS boundaries could not be clearly chosen due to intraconal glioma, these parameters of the eyes were not included in the statistical analyses of the study. 

Optic nerve tortuosity was assessed by the neuroradiologist (M.B.) and graded as follows:

Grade 0: No tortuosity

Grade 1: Mild tortuosity

Grade 2: Moderate tortuosity

Grade 3: Severe tortuosity

The optic pathways including optic radiations were also evaluated for the presence of any NBO, hamartoma, myelin defect, axonal defect, or OPG. Gliomas of the optic pathways were defined based on hyperintense appearance (on FLAIR or T2W images) and thickening of optic pathway structures [8]. The location (prechiasmatic, chiasmatic, postchiasmatic) and the laterality (right-sided, left-sided, bilateral) of the glioma in patients with OPG were classified. Findings of STIR and 3D-SPACE sequences were compared between groups and according to clinical findings. 

### 2.1. Statistical analysis

Continuous variables were reported with median and minimum–maximum values, and categorical variables were presented as frequency and percentage. Kruskal–Wallis and Mann–Whitney U tests were performed for group comparisons. Categorical variables were compared between groups using Pearson’s chi-square test, Fisher’s exact test, and the Fisher–Freeman–Halton test. For determining the reliability of 3D-SPACE and STIR measurements, the intraclass correlation coefficient (ICC) was used. Statistical signiﬁcance was set at P < 0.05. 

## 3. Results 

Group 1 consisted of 20 patients. There were 10 males and 10 females with a mean age of 17.8 (7–41) years. Group 2 comprised 16 patients including 7 (43.8%) males and 9 (56.2%) females. Their mean age was 12 (6–21) years. The OPGs were right-sided in 4 (25%), left-sided in 2 (12.5%), and bilateral in 10 (62.5%) patients in Group 2. The control group consisted of 19 subjects, 9 (47.4%) males and 10 (52.6%) females, with a mean age of 18.3 (5–34) years. 

The mean ONS diameters on STIR images were significantly higher in Group 2 compared to Group 1 and controls (p=0.009) (Table 2). The mean ONS diameter was significantly higher in Group 2 when compared to Group 1 and the control group with the 3D-SPACE sequence (p=0.017, Table 2). It was 4.4 mm (3.3–6.3), 5.4 mm (3.2–14.5), and 5.1 mm (4.2–6.5) in Group 1, Group 2, and the control group, respectively (p=0.019 for Groups 1 and 2, p=0.007 for Group 1 and control group, p=0.607 for Group 2 and control group) (Table 2). 

**Table 2:** The median thickness of the optic nerve (ON) and the diameter of the optic nerve sheath (ONS) measured with two different MRI sequences in groups.

MRI Sequence	Group 1 Median (min–max)	Group 2 Median (min–max)	Controls Median (min–max)	p
ONS-SPACE (mm)	4.4 (3.3–6.3)	5.4 (3.2–14.5)	5.1 (4.2–6.5)	0.017
ON-SPACE (mm)	2.3 (1.5–3.5)	2.5 (1.4–34)	2.4 (1.7–3.7)	0.193
ONS-STIR (mm)	5.3 (4.3–7.3)	6.7 (4.4–14)	5.7 (5–7.3)	0.009
ON-STIR (mm)	3.2 (2.3–4.4)	3 (1.7–10)	2.8 (2.1–3.8)	0.804

Group 1: NF-1 patients without optic pathway glioma (OPG); Group 2: NF-1 patients with OPG.

Measurements of ONS diameter obtained from STIR and 3D-SPACE sequences were significantly correlated with each other (p<0.001, ICC: 0.88, Table 3). Comparing the distribution of the ON tortuosity grades across the controls and Groups 1 and 2 (Table 4), control subjects had significantly lower grades than both patient groups (p=0.001). Group 1 also had significantly lower tortuosity grades when compared to Group 2 (p=0.001). Severe (grade 3) tortuosity was only detected in Group 2. 

**Table 3:** Compatibility and correlation analyses of the two methods (3D-SPACE and STIR) for thickness of the optic nerve (ON) and diameter of the optic nerve sheath (ONS).

	Group 1		Group 2		Controls		Overall	
	ICC	P	ICC	P	ICC	P	ICC	p
ON	-	0.396	-	0.196	-	0.946	-	0.082
ONS	0.94	<0.001	0.95	<0.001	-	0.806	0.88	<0.001

ICC: Intraclass correlation coefficient, Group 1: NF-1 patients without optic pathway glioma (OPG), Group 2: NF-1 patients with OPG.

**Table 4:** The grades of optic nerve tortuosity in groups.

Grade	Group 1# (%)	Group 2# (%)	Controls# (%)	Total# (%)
0	6 (30)	2 (7.7)	17 (89.5)	25 (38.5)
1	9 (45)	9 (34.6)	2 (10.5)	20 (30.8)
2	5 (25)	4 (15.4)	0 (0)	9 (13.8)
3	0 (0)	11 (42.3)	0 (0)	11 (16.9)

Group 1: NF-1 patients without optic pathway glioma (OPG); Group 2: NF-1 patients with OPG.

In Group 1 and Group 2, 7 (35%) and 5 (31.2%) patients had a positive family history of NF-1, respectively. Seven (35%) patients in Group 1 and 3 (18.7%) patients in Group 2 had Lisch nodules. There was no association between the presence of any OPG and having a positive family history, presence of any Lisch nodule, or hydrocephalus (P > 0.05). Patients with headaches had higher (but not statistically significant) ON thickness and ONS diameter when compared to patients without headaches (Table 5).

**Table 5:** Relationship between the thickness of the optic nerve (ON) and optic nerve sheath (ONS) diameter with the presence of headache in the whole NF-1 patient cohort.

Sequence	ON/ONS	No Headache	Headache	P-value
		Median (min–max)	Median (min–max)	
STIR	ON	2.90 (1.70–4.20)	3.35 (2.50–5.30)	0.182
	ONS	5.70 (4.30–8.40)	6.10 (4.80–8.50)	0.457
3D-SPACE	ON	2.40 (1.50–34)	2.40 (1.80–4.90)	0.585
	ONS	5 (3.20–6.50)	5.10 (3.50–8)	0.499

**Figure 2:**
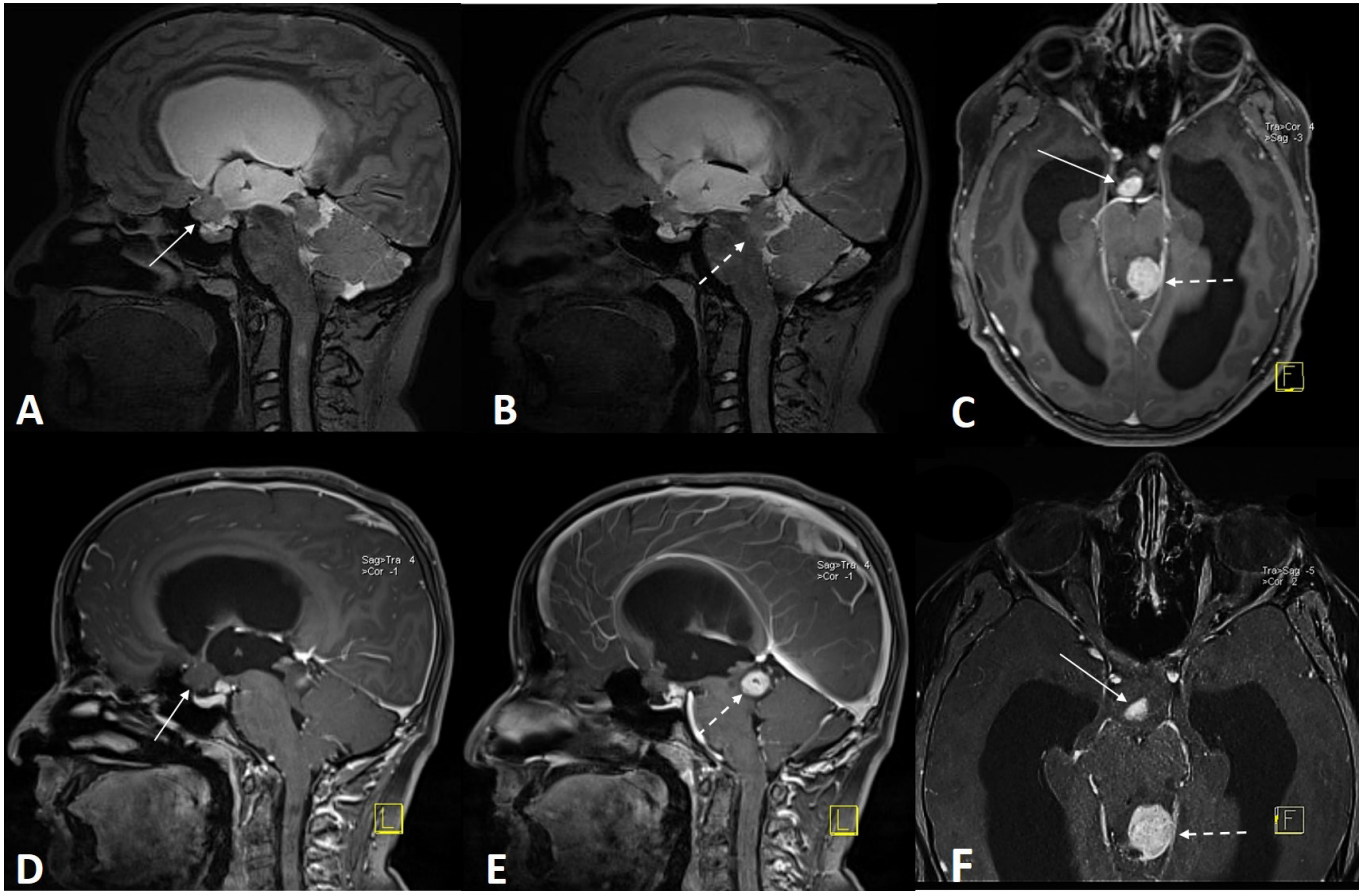
3D-SPACE (T2W with variant FA mode) (A, B) and contrast-material enhanced MPRAGE (C–F) images of an NF-1 patient with optic pathway glioma (arrows) and aqueduct stenosis due to tectal glioma (dashed arrow).

Partial or complete type aqueduct stenosis was detected in six of NF-1 patients, of whom three had hydrocephalus (two patients in Group 1 and one patient in Group 2) and the other three had functioning ventriculoperitoneal shunt systems (Figure 2). Cranial MRI findings (except NBOs or gliomas) of all NF-1 patients are given in Table 6. In total, 109 NBOs were detected in Group 1 and 97 were detected in Group 2 with the STIR sequence imaging. 3D-SPACE imaging demonstrated an additional 26 NBOs in Group 1 and 17 NBOs in Group 2 patients.

**Table 6:** Cranial MRI findings (except unidentified bright objects) of NF-1 patients in Groups 1 and 2.

Group 1	Number of patients	Number of pathologies
Hyperintense bone lesion on T2W images	1	1
Mucosal thickening of the paranasal sinuses	5	13
Cavum septum pellucidum	1	1
Abnormal course of the optic nerve	1	1
Adenoid vegetation	4	4 (bilaterally)
Pineal cyst	1	1
Encephalomalacia due to trauma or surgery	1	1
Intracranial cystic lesion (e.g., arachnoid cyst)	2	3
Intracranial hypotension	1	1
Group 2	Number of patients	Number of pathologies
Nonoptic pathway glioma	4	5
Mucosal thickening of the paranasal sinuses	7	16
Adenoid vegetation	4	4 (bilaterally)
Encephalomalacia due to trauma or surgery	1	1
Contrast-material enhanced glioma	4	4
Hyperintense bone lesion on T2W images	1	2
Hypophyseal adenoma	1	1
Mega cisterna magna	1	1

Group 1: NF-1 patients without optic pathway glioma (OPG), Group 2: NF-1 patients with OPG

## 4. Discussion

This paper focuses on two main topics: the description of the optic nerve in patients with NF-1, and the comparison of NF-1-related findings on routinely performed MRI sequences (2D-STIR) versus upcoming high-resolution 3D-MRI acquisition (3D-SPACE). Patients with OPGs are at risk for vision loss. Optic pathway gliomas are one of the seven diagnostic criteria of NF-1 [1–8]. Ophthalmologic examination may show optic nerve dysfunction. Dilatation of the ONS and tortuosity of ON are other common MRI findings in NF-1 patients [12–14]. ONS dilatation is the nonprogressive enlargement of the subarachnoid space surrounding the optic nerve, synonymous with dural ectasia of the ONS [15]. ONS dilatation is a sign, not a disease, and can easily be detected and measured with MRI and CT scans. It is caused by underlying processes, which themselves may need treatment, but ONS dilatation itself does not need treatment. 

Recent research indicates that ONS dilatation could be a good indicator of increased intracranial pressure (ICP) in NF-1 patients [14]. Therefore, ONS fenestration, a surgical procedure to reduce the pressure within the subarachnoid space of the optic nerve, could be helpful in the treatment of severe ONS dilatation associated with progressive visual loss due to papilledema [14–16]. Optic nerve sheath dilatation can occur for other reasons unrelated to high ICP and these cases are unlikely to benefit from ONS fenestration. 

Increased ON tortuosity has been demonstrated in the optic pathway of NF-1 patients in a few reports [8,12–14]. In a recent study, the incidence of ON tortuosity was reported as 84% in NF-1 patients [17]. The two most reliable features of ON tortuosity were lack of congruity in greater than one coronal section and dilatation of the subarachnoid space surrounding the anterior portion of the ON. Levin et al.’s study revealed that ON tortuosity on baseline MRI was a predictor of subsequent OPG in NF-1 patients, but not a predictor of clinically significant OPG requiring treatment or resulting in poor vision. ON thickness and ONS dilatation were not associated with later appearance of OPG [8].

In the present study, we evaluated the optic pathways of NF-1 patients with a 3T-MRI unit. We assessed and compared the thickness and the tortuosity of the ON and the diameter of the ONS in patients with or without OPG by 3D-SPACE and STIR sequences. Our study demonstrated that the diameter of the ONS was significantly higher in NF-1 patients regardless of the presence of OPG. The ON thickness did not show any significant difference between NF-1 patients and healthy subjects. We think that this result is related to our measurement location because we measured the ON diameter apart from the visible OPG in NF-1 patients with OPG. The aim of this was to evaluate the normal-appearing parts of the ONs.

The relationship between raised intracranial pressure and the dilatation of the ONS has been known for several years [15–18]. Also, it is known that NF-1 is associated with altered or worsened CSF circulation due to fibrotic changes of the arachnoid membranes [19]. Mild or moderate intracranial hypertension might be present in NF-1 patients regardless of having OPG. Supporting this hypothesis, we observed that headache was more common in NF-1 patients with more dilatated ONS. According to our data, ONS diameter will not relate solely or principally to the presence or absence of an OPG. In many cases, the ONS dilatates secondary to the intracranial phenomena. We think that studies monitoring the pressure of cerebrospinal fluid are needed to support our findings. 

ON tortuosity was assessed subjectively in our study. Our findings revealed that the average ON tortuosity for NF-1 patients with OPG was higher than that of the NF-1 patients without OPG. NF-1 patients without OPG also had more tortuous ONs when compared to healthy subjects. Grade 3 ON tortuosity was only detected in NF-1 patients with OPG, while healthy subjects had none. We think that increased ON tortuosity in NF-1 patients may also indicate raised intracranial pressure and support our previous findings. 

Additionally, our results indicated that both 3D-SPACE and STIR sequences provide valuable imaging information on the diameter of the ONS. Thin-section conventional sequences would be preferable in evaluating the morphology of the ON or other cranium structures of the NF-1 patients [9]. Isotropic 3D-SPACE (<1 mm3) data are useful for evaluation of these details and have complementary capability for multiplanar high-resolution CSF and whole cranium evaluation, but this needs more experience. Importantly, 3D-SPACE and conventional sequences for assessment of optic pathway structures seem to complement each other and give partially different information. 3D-SPACE imaging provides information to be of potential use in daily clinical practice in easy screening of the optic pathway and the whole cranium with high resolution and low SAR levels [11].

The relationship between CSF-related disorders (especially hydrocephalus) and NF-1 has already been reported [9–19]. In our study, the 3D-SPACE sequence was adequate for imaging of our patients with hydrocephalus or ventriculoperitoneal shunt and their other intracranial findings. In the literature, the 3D-SPACE technique was reported as the easiest, fastest, and most adequate sequence to determine the ventricular system and CSF-related pathologies [20,21].

There are many T2W 3D-SPACE or 3D-TSE sequences for evaluation of the cranium or brain [20]. The 3D-SPACE sequence of this study is not a heavily T2-weighted sequence. It is a 3D-turbo spin echo (3D-TSE) sequence with variant FA values [21]. According to our observations, the increased signal intensity of some lesions (e.g., an important feature of optic nerve gliomas) may not be evaluated perfectly on 3D-SPACE images. From a radiologist’s perspective, in the absence of other findings, the tortuosity of the ON may cause confusion among clinicians about the presence of optic nerve glioma in the patient. The point is that the ON tortuosity is not a unique indicator of the presence of optic pathway gliomas.

According to the literature, the upper limit or threshold value for ONS diameter is 6 mm in healthy children and adolescents [22]. Our findings are consistent with these statements. Median ONS values of our control group for 3D-SPACE and STIR sequences are 5.1 and 5.7 mm, respectively.

In contrast with the ONS diameter, ON measurements were not correlated between the 3D-SPACE and STIR sequences (Table 3). These results are probably due to the fact that 3D-SPACE with variant or variable FA technique is more sensitive to the delineation of intraconal ON than the STIR images. On these types of 3D-SPACE images, intraconal ON visibility is lesser than in STIR images because STIR is a fluid-sensitive, fat-saturated, and relatively heavily T2W technique. 3D-SPACE or 3D-TSE images with constant FA values may be more useful for delineation of ON/ONS and comparison of these values with STIR sequence.

There are some limitations to our study. First, monitoring with MRI and clinical exams frequently decreases dramatically after age 8. Furthermore, OPGs are known to regress into adulthood. Since sedation was needed, we could not include more pediatric cases in the study groups. Second, the proof or exclusion of an OPG was subjectively determined due to ethical reasons. Enlargement of the optic nerve in the setting of NF-1 is assumed to be a sign of OPG and biopsy confirmation is generally not required. Contrast-material enhancement on T1W images suggests more active disease, but OPG is suspected even in nonenhancing slight expansion of the optic nerve. However, the exact diagnosis of the OPG is made by biopsy. Third, the cross-section of the optic nerve is around 3 mm in all three groups. The voxel sizes of 3D-SPACE and 2D-STIR sequences are 0.6 × 0.6 × 0.6 and 0.6 × 0.5 × 3 mm, respectively. These values may be inadequate for optic nerves, and subtle size differences are within the margin of error (especially for STIR images). Fourth, this is a small study in which STIR statistically performed slightly better than 3D-SPACE imaging in assessing ON thickness. Also, STIR can offer an advantage in terms of assessment of the optic nerve signal. Finally, interrater and intrarater variability assessments would be helpful for optimal detection of possible differences between sequences and other parameters.

In conclusion, our findings revealed that the ON is more tortuous and ONS is more dilatated in NF-1 patients (especially in cases with OPG). Additionally, high-resolution 3D cranium imaging with 3D-SPACE is useful for the management of patients with NF-1. The 3D-SPACE technique is safe, efficient, and relatively easy in assessing the optic pathways in NF-1 patients. In the current study, we apparently demonstrated the equivalency of T2W 3D-SPACE versus STIR for measurement of the ON and ONS in the NF-1 population and controls. Further studies with large patient groups are needed to support our findings. 
